# Effects of birth weight and growth traits on the first lactation milk yield and reproduction characteristics in Anatolian buffaloes

**DOI:** 10.5194/aab-68-193-2025

**Published:** 2025-03-17

**Authors:** Sinem Akçam, Ertuğrul Kul

**Affiliations:** 1 Department of Animal Science, Institute of Natural and Applied Sciences, University of Kırşehir Ahi Evran, Kırşehir, Türkiye; 2 Department of Animal Science, Faculty of Agriculture, University of Kırşehir Ahi Evran, Kırşehir, Türkiye

## Abstract

This study aimed to reveal the effects of birth weight (BW), 6-month live weight (LW_6_), and 12-month LW (LW_12_) on first lactation milk yield and reproduction characteristics and to determine the non-genetic factors affecting these traits of Anatolian buffaloes in Amasya Province. For this purpose, 200 Anatolian buffaloes born between 2014 and 2018 and calved between 2017 and 2021 formed the basis of this study. These animals were divided into two groups with respect to BW ( <26.82 and ≥26.82 kg), LW_6_ ( <88.00 and ≥88.00 kg), and LW_12_ ( <138.47 and ≥138.47 kg). The effect of birth year on BW ( P=0.044 ), LW_6_ ( P<0.001 ), and LW_12_ ( P<0.001 ) was statistically significant, while there was no significant effect of birth season on BW and LW_6_ ( P>0.05 ), except LW_12_ ( P<0.001 ). The effect of dam age on LW_6_ was significant ( P=0.039 ), except BW and LW_12_ ( P>0.05 ). In the present study, only the BW groups had a significant effect on lactation length (LL) ( P=0.006 ). The effect of growth traits on the first calving age (FCA) of the LW_12_ group was significant ( P=0.036 ). Although it was not statistically significant, it can be said that buffaloes with higher LW in terms of BW and growth traits had higher lactation milk yield (LMY), earlier FCA, and shorter calving intervals (CIs). There have been limited studies on the effects of BW and growth traits on milk and reproduction traits in buffaloes, and most of the studies were conducted in cattle. Therefore, more studies are required to reveal the relationships between related traits in buffaloes.

## 1 Introduction

The presence of buffaloes in Türkiye dates back to 3000 BCE (Alkoyak and Öz, 2022). The buffaloes raised in Türkiye, known as Anatolian buffaloes, originate from Mediterranean buffaloes, a subgroup of riverine buffaloes (Kul et al., 2016; Soysal et al., 2018). Buffaloes in Türkiye are bred for milk and meat production (Atasever and Erdem, 2008). In addition to their resistance to diseases and harsh environmental conditions, buffaloes are valued for their efficiency in converting low-quality feed into high-quality milk and meat, as well as for their lower husbandry costs compared to dairy cattle (Atasever and Erdem, 2008; Kul et al., 2016). These animals are typically black or dark gray with horns that curl upward, bending backward or to the sides (Soysal et al., 2018; Koçak et al., 2019). Buffaloes are distributed across Türkiye, where the majority of the national population resides (Alkoyak and Öz, 2022). The water buffalo population in Türkiye was an important genetic resource with approximately 161 749 heads in 2023 (TUIK, 2024). Buffalo breeding in Türkiye is predominantly practiced in small-scale family farms, with a few larger commercial operations (Koçak et al., 2019).

In the continuity of a herd, the age of the heifer has an impact on their subsequent reproduction and milk yield performance. In this respect, the process from birth to the first calf should be well managed (Göncü, 2020). In this manner, the herd size will be maintained, and genetically superior heifers will be selected for the continuation of the herd (Mourits et al., 1999). Minimizing calf mortalities, which result in production losses, and increasing the growth performance of young animals that create high vitality and future generations always remain important for economical production (Yüceer and Özbeyaz, 2010). Buffalo heifers gain faster to begin their physiological processes earlier for reproduction and milk production (Yadav et al., 2001). Therefore, increasing milk and fertility performance are closely related to the growth performance of young animals from birth to adulthood.

Birth weight (BW), which is the first observable polygenic characteristic of the animal, is the first phenotypic indicator of the genotype and is a critical feature for subsequent growth (Yadav et al., 2001). In addition to the BW of the buffalo, its growth rate also plays a significant role in determining its health and production traits in later years. Understanding the relationships between calf growth characteristics and future productivity and identifying influencing factors will facilitate the development of the best herd management strategies to optimize calf growth and to select heifers based on early-life measurements (Wathes et al., 2008). The BW of buffaloes is affected by non-genetic factors such as dam age (Thiruvenkadan et al., 2009), season (Hossein-Zadeh et al., 2012), and year (Akyol, 2023). The animal's actual genetic potential for growth may be suppressed by these factors.

High BW positively affects subsequent lactation milk yield (LMY; Hoffman, 1997). However, increasing the feeding level during the breeding period causes a decrease in subsequent milk yield by causing fat accumulation rather than skeletal and epithelia tissue growth due to heavier heifers at first calving, negatively affecting udder development (Mourits et al., 2000). Because mammary gland development mostly occurs before calving, a younger calving age also leads to reduced milk yield due to poor mammary tissue development (Serjsen, 2005).

The obtained data regarding the effects of BW, growth characteristics, and first calving age (FCA) on the subsequent performance of buffaloes have been incompatible. In addition, the number of studies revealing the effects of BW and growth characteristics on productivity traits has been quite limited. These kinds of studies were mostly conducted in cattle. In the present study, we aimed to reveal the effects of BW and 6-month live weight (LW_6_) and 12-month LW (LW_12_) of Anatolian buffaloes on the first LMY and reproduction traits and to determine the effects of non-genetic factors on these traits.

## 2 Materials and methods

This study was conducted with 200 Anatolian buffaloes born from 2014 to 2018 and calved from 2017 to 2021 within the scope of the Public Anatolian Buffalo Breeding Project conducted in Amasya Province.

The birth date, sex, dam number, and other identifying information of the calves were recorded within 24 h by using the Buffalo Star recording program. The weights of the calves were measured on the day they were born by using a hand scale. After the calves were marked with ear tags, all data were recorded by using the Buffalo Star software developed for the Public Anatolian Buffalo Breeding Project (Tekerli, 2015–2018). The LW_6_ and LW_12_ of calves were determined and recorded to compute LW_6_ and LW_12_ using linear interpolation.

Monthly milk yields of all buffaloes were recorded during lactation. Daily milk yield (DMY), lactation milk yield (LMY), and lactation length (LL) were obtained. During the study, data regarding calving date, first calving age (FCA), and second calving age were entered into the this program to calculate the FCA, calving interval (CI), and dry period (DP).

Effects of birth year, birth season, and dam age on BW, LW_6_, and LW_12_ were evaluated by using the following linear model:

1 $$Y_{ijkl}=\mu +a_{i}+b_{j}+c_{k}+e_{ijkl},$$

where $Y_{ijkl}$ is the dependable variable, μ the overall mean, $\alpha_{i}$ the effect of birth year ( i : 2014, 2015, 2016, 2017, 2018), $b_{j}$ the j effect of birth season ( j : winter, spring, summer, autumn), $c_{k}$ the k effect of dam age ( k : younger: 2, 3, 4; moderate: 5, 6, 7; older: 8, 9, ≥10 ages), and $e_{ijkl}$ the random error.

Groupings for BW, LW_6_, and LW_12_ were based on their means. The first group was below the average; the second group was above the average. To evaluate the effects of BW, LW_6_, and LW_12_ on milk yield and reproduction traits, the following linear model was applied:

2 $$Y_{ijkl}=\mu +a_{i}+b_{j}+c_{k}+e_{ijkl},$$

where $Y_{ijkl}$ is the dependable variable, μ the overall mean, $\alpha_{i}$ the i effect of BW ( i : <26.82 kg, ≥26.82 kg), $b_{j}$ the j effect of LW_6_ ( j : <88.00 kg, ≥88.00 kg), $c_{k}$ the k effect of LW_12_ ( k : <138.47 kg, ≥138.47 kg), and $e_{ijkl}$ the random error.

Normality analyses performed with Shapiro–Wilk and Kolmogorov–Smirnov tests satisfied the assumptions ( P>0.05 ). Furthermore, the homogeneity of the variances was established by the Levene test ( P>0.05 ). In the study, a general linear model analysis was used to statistically compare three groups, and a t test was used to compare two groups. Differences between subgroups were compared using Duncan's multiple-range test. In addition, Pearson correlation coefficients were calculated in the study. The data were analyzed using SPSS 17 for Windows.

## 3 Results and discussions

The effects of birth year, birth season, and dam age on BW, LW_6_, and LW_12_ are given in Table 1. The effect of birth year was significant on BW ( P=0.044 ), LW_6_ ( P<0.001 ), and LW_12_ ( P<0.001 ). The highest BW of calves was determined in 2017 and the lowest in 2014. There was no significant difference between the BWs of those whose birth years were 2015, 2016, and 2018. The highest LW_6_ and LW_12_ were observed in 2016 ( P<0.001 ). There was no significant difference between the LW_6_ data, except for 2016. The lowest LW_12_ was determined in 2014 and 2015. In this study, the variations in BW and growth characteristics across different years reflect the influence of farm management practices and environmental conditions. Animals born later in the data collection period exhibited higher body weights, suggesting improved farm management over time. Management quality often varies with factors such as farmer expertise, farming methods, selection criteria, and stocking density (Alkoyak and Öz, 2022). The year of birth effect is likely attributable to differences in herd size, environmental factors, management strategies, fodder availability, and breeding plans (Sadek et al., 2014). These findings were expected, given that pasture-based feeding in rural settings depends heavily on climatic conditions affecting pasture growth and feed availability. Similar results were reported for BW in buffaloes (Thiruvenkadan et al., 2009; Hossein-Zadeh et al., 2012; Akyol, 2023), LW_6_ (Thiruvenkadan et al., 2009; Akyol, 2023), and LW_12_ (Akyol, 2023).

**Table 1:** Effects of non-genetic factors on BW, LW_6_, and LW_12_ (mean ± SE).

		N	BW	N	LW_6_	N	LW_12_
Birth year	P value		0.044		<0.001		<0.001
	2014	39	$25.21\pm 0.61^{b}$	42	$88.47\pm 2.90^{b}$	42	$129.74\pm 3.44^{c}$
	2015	51	$26.97\pm 0.73^{\text{ab}}$	51	$82.19\pm 1.71^{b}$	49	$130.77\pm 2.12^{c}$
	2016	41	$27.42\pm 0.71^{\text{ab}}$	42	$102.53\pm 4.09^{a}$	42	$153.89\pm 4.01^{a}$
	2017	35	$27.83\pm 1.04^{a}$	35	$82.70\pm 3.20^{b}$	35	$141.30\pm 3.80^{b}$
	2018	30	$26.67\pm 0.72^{\text{ab}}$	30	$83.08\pm 1.76^{b}$	30	$138.39\pm 2.26^{\text{bc}}$
Birth season	P value		0.065		0.348		<0.001
	Winter	31	26.55±0.86	34	86.27±2.80	33	$135.95\pm 3.04^{b}$
	Spring	72	26.04±0.61	74	87.25±2.31	74	$131.70\pm 2.32^{b}$
	Summer	78	27.92±0.52	77	90.71±2.51	76	$146.50\pm 2.84^{a}$
	Autumn	15	25.42±1.02	15	81.74±3.33	15	$136.79\pm 3.96^{b}$
Dam age	P value		0.194		0.039		0.162
	1 (2–4 ages)	51	27.61±0.62	53	$93.38\pm 3.10^{a}$	53	140.50±3.41
	2 (5–7 ages)	83	26.97±0.56	85	$85.05\pm 1.83^{b}$	83	134.96±2.16
	3 (8– ≥ 10 ages)	62	25.98±0.62	62	$87.45\pm 2.58^{\text{ab}}$	62	141.44±2.86
Overall		196	26.82±0.35	200	88.00±1.40	198	138.47±1.57

^a,b,c^ Different superscripts in the same column denote significance ( P<0.05 ). BW: birth weight; LW_6_: 6-month live weight; LW_12_: 12-month live weight.

There were conflicting results, indicating that the birth year had no effect on the BW of Egyptian buffalo (Marai et al., 2009) and on the LW_12_ of Surti buffalo (Pandya et al., 2015).

In the current study, while there was no significant effect of birth season on BW ( P=0.065 ) and LW_6_ ( P=0.348 ), LW_12_ was affected by birth season ( P<0.001 ). The highest LW_12_ was determined in calves born in the summer, but no significant difference was determined between the LW_12_ of those born in other seasons. Although the differences were not statistically significant, calves born in the summer season, whose mothers grazed on spring–summer pastures during the late stages of pregnancy and the nursing period, exhibited higher BW and LW_6_. Calves born in the summer also had higher LW_12_, likely due to grazing directly on spring and summer pastures post-weaning. This finding can be attributed to favorable temperatures and the availability of high-quality green fodder during these seasons (Uğurlu et al., 2016). As expected, the rainy season provides more abundant and higher-quality pasture, enhancing growth traits in buffaloes (Segura-Correa et al., 2017). In Amasya Province, buffaloes are kept indoors and tied during winter, preventing winter-born buffaloes from grazing (Koçak et al., 2019). Additionally, pasture quality declines during autumn, a transitional period (Kul et al., 2018). The results obtained in this study were similar to those of previous studies on BW (Thiruvenkadan et al., 2009; Akkulak and Kul, 2023), LW_6_ (Thiruvenkadan et al., 2009), and LW_12_ (Akkulak and Kul, 2023) in buffaloes. In detail, the birth season had significant effects on BW (Hossein-Zadeh et al., 2012; Kul et al., 2018) and LW_6_ (Akkulak and Kul, 2023) in buffaloes. Thiruvenkadan et al. (2009) reported that the LW_12_ in Murrah buffaloes was not affected by the season of birth, unlike the current study. Erdem et al. (2015) reported that both BW and LW_6_ of Anatolian buffaloes were the highest in autumn ( P<0.001 ). Marai et al. (2009) observed the lowest BW in Egyptian buffalo calves born in summer. Kul et al. (2018) determined the lowest BW in Anatolian buffalo calves born in autumn and the highest in summer.

Dam age affected LW_6_ significantly ( P=0.039 ) but not BW ( P=0.194 ) and LW_12_ ( P=0.162 ). LW_6_ was observed to be the highest in the calves of younger dams in the first age group but the lowest in the second age group (Table 1). The present results are similar to those of Erdem et al. (2015). These results can be explained by the fact that young buffaloes, still growing towards their adult size, may further the development of the fetus during pregnancy (Kul et al., 2018). However, the selection of young buffaloes with superior growth traits for the purpose of projects, their heavier calves, and improvements in the care and feeding practices at farms are believed to have influenced these outcomes. However, this finding disagrees with those of Hossein-Zadeh et al. (2012) and Kul et al. (2018) in that the effect of dam age on BW was significant. This finding was not consistent with the results of Erdem et al. (2015), who reported that the effect of dam age on LW_12_ in Anatolian buffaloes was significant. Hossein-Zadeh et al. (2012) determined that older dams had heavier buffalo calves. In our study, without statistical significance, the BW, LW_6_, and LW_12_ of the calves born from younger dams were higher.

In buffaloes, LMY and LL are important indicators of their genetic potential (Verma et al., 2021). A DP is crucial for buffaloes as it allows the animal to rest and repair udder tissue, thereby preparing for the next lactation. This rest period is essential for maximizing milk production in the following lactation cycle (Eldawy et al., 2021). As shown in Table 2, the BW of calves had a significant effect on the LL ( P=0.006 ), whereas other milk yield traits were not affected by growth traits. These findings demonstrate that LL increased with increasing BW. Although it was not statistically significant ( P>0.05 ), it was seen that the LMY values of calves with higher BW, LW_6_, and LW_12_ were higher than those of lighter ones.

**Table 2:** Changes in milk yield and reproduction traits according to BW, LW_6_, and LW_12_ groups (mean ± SE).

		Milk yield traits								Reproduction traits			
		N	LMY	N	LL	N	DMY	N	DP	N	FCA	N	CI
			(kg)		(d)		(kg)		(d)		(month)		(d)
BW	P value		0.130		0.006		0.914		0.110		0.369		0.085
	1 ( <26.82 kg)	103	918.55±15.15	103	$209.07\pm 2.14^{b}$	103	4.40±0.06	21	250.43±28.68	110	42.92±1.24	21	443.05±25.86
	2 ( ≥26.82 kg)	78	953.35±15.98	78	$218.35\pm 2.45^{a}$	77	4.39±0.07	20	212.30±24.53	86	42.10±1.27	20	432.55±23.26
LW_6_	P value		0.585		0.755		0.203		0.080		0.345		0.183
	1 ( <88.00 kg)	113	930.09±13.90	113	214.64±2.01	113	4.34±0.06	24	227.96±27.82	123	42.36±1.03	24	434.96±24.09
	2 ( ≥88.00 kg)	72	939.33±17.69	72	210.93±2.76	71	4.48±0.08	18	218.33±24.77	77	43.68±1.63	18	427.17±24.36
LW_12_	P value		0.271		0.549		0.556		0.398		0.036		0.296
	1 ( <138.47 kg)	116	918.79±13.21	116	211.58±2.07	115	4.36±0.06	25	233.16±28.78	124	$44.08\pm 1.21^{a}$	25	442.76±26.72
	2 ( ≥138.47 kg)	67	953.44±18.87	67	215.21±2.68	67	4.44±0.08	16	209.94±21.75	74	$40.51\pm 1.24^{b}$	16	413.38±16.53

^a, b^ Different superscripts in the same column denote statistical significance ( P<0.05 ). LMY: lactation milk yield; LL: lactation length; DMY: daily milk yield; DP: dry period. BW: birth weight; LW_6_: 6-month live weight; LW_12_: 12-month live weight.

According to several studies, growth rate in the early stages of life has a positive effect on subsequent milk yield (Hoffman, 1997). Carson et al. (2002) determined that heavier Holstein Friesian heifers before the first calving had higher milk yield. This can be explained by the better development of udder tissue in heavier heifers (Serjsen, 2005). The results obtained in this study are similar to those obtained by Heinrichs and Heinrichs (2011), who reported that dairy calves with a faster growth had higher LMY at the first lactation. In contrast to these results, Mourits et al. (2000) indicated that more fat accumulation in the udder tissue of heavier dairy heifers adversely affects udder development and leads to a decrease in subsequent milk yield. The results of this study are different from those of other studies for the following reasons: the studies were conducted on different breeds in different countries and regions, and there were differences in herd management practices between dairy farms.

As seen in Table 2, the LW_12_ group had a significant effect on FCA ( P=0.036 ), but the CI was not affected by any of growth traits ( P=0.296 ). In addition, the FCA was calculated to be higher in buffaloes with a lighter LW_12_ (first group). The growth performance of buffaloes raised by smallholder farmers is a critical and vulnerable factor (Bayou et al., 2015). Low live weights during early lactation can decrease the likelihood of pregnancy at the first insemination due to insufficient energy needed for ovarian activity and estrus (Eldawy et al., 2021). Additionally, lighter Holstein heifers reach puberty later than heavier ones (Sadek et al., 2014). Heavier heifers likely have more mature reproductive tracts due to alternating estradiol and progesterone peaks after reaching puberty (López et al., 2018). These findings suggest that achieving adequate skeletal maturity through proper early growth is essential for successful calving in heifers (Cooke et al., 2013). One possible explanation for discrepancies in growth rates, associated with reduced FCA and CI, is physiological immaturity at the time of first breeding (Brickell et al., 2009). Further research is needed to confirm the impact of BW and early growth on subsequent fertility and productivity.

The CI has great economic importance and is closely related to LMY and LL (Verma et al., 2021). Shortening the first CI will contribute to shortening the generation interval and increasing the genetic progress of the herd (Muasya et al., 2013). It will also reduce management costs such as feed and labor (Tozer and Heinrichs, 2001).

FCA is a crucial factor in determining the length of a nonproductive period and influencing subsequent fertility (López at al., 2018). FCA is strongly influenced by the growth rate, which generally has high heritability in Italian Holsteins (Verma et al., 2021). Pirlo et al. (2000) reported that a decrease in the FCA adversely affected the first LMY in Italian Holsteins, associated with low BW in young heifers at the beginning of the first lactation. Boopathi et al. (2021) found that BW did not significantly affect reproductive performance in Murrah buffaloes. Eldawy et al. (2021) determined that the effects of BW on the FCA and CI of Egyptian buffaloes were significant ( P<0.05 ). Akbulut et al. (1998) found that the FCA was significantly affected by BW, LW_6_, and LW_12_ in Brown Swiss cattle ( P<0.05 ). Ettema and Santos (2004) did not see the effect of FCA on subsequent reproductive performance of Holstein cows, and more studies need to be conducted on this subject. This difference may be attributed to various management and environmental conditions, herd and farm sizes, variations in feed and fodder availability, and the sires used for breeding and their genetic potential (Kul et al., 2018).

The correlation analysis (Table 3) showed that there was a positively low correlation between BW and milk yield characteristics ( 0.026≤r≤0.134 ; P>0.05 ), except for the DP ( r=-0.146 , P>0.05 ). According to the findings obtained in this study, the correlation between the LW_12_ and FCA was negative (with statistical significance r=-0.148 , P=0.037 ), whereas there was an insignificant correlation between LW_6_ and FCA ( r=0.002 , P>0.05 ). Positive correlation between BW and LMY ( r=0.331 ) in Anatolian buffaloes was observed by Akkulak and Kul (2023). In the same study, correlations between LW_6_ with LMY ( r=0.267 ) and DMY ( r=0.339 ) were found to be positive. Yıldız et al. (2008) determined a negative and significant correlation between BW and LMY in Eastern Anatolian Red cows ( P<0.05 ). Akbulut et al. (1998) found insignificant negative correlations between 305 d milk yield and BW and LW_12_ and positive correlations with LW_6_ in Brown Swiss cattle. Sorathiya et al. (2009) found negative correlations between the BW and FCA and between LW_6_ and LW_12_ in Surti buffaloes. Naqvi and Shami (1999) did not determine any significant relationship between BW and the FCA of dams in Nili-Ravi buffalo calves ( P>0.05 ).

**Table 3:** Correlations among BW, LW_6_, and LW_12_ with milk yield and reproduction traits.

	Milk yield traits				Reproduction traits	
	LMY	LL	DMY	DP	FCA	CI
	(kg)	(d)	(kg)	(d)	(month)	(d)
BW	0.108	0.134	0.026	-0.146	-0.088	-0.047
	(0.147)	(0.071)	(0.724)	(0.362)	(0.222)	(0.773)
LW_6_	-0.006	-0.099	0.079	-0.210	0.002	-0.210
	(0.934)	(0.180)	(0.288)	(0.181)	(0.978)	(0.182)
LW_12_	0.057	0.077	0.009	-0.132	-0.148	-0.137
	(0.445)	(0.301)	(0.908)	(0.409)	(0.037)	(0.393)

LMY: lactation milk yield; LL: lactation length; DMY: daily milk yield; DP: dry period; FCA: first calving age; CI: calving interval; LW: live weight; BW: birth weight; LW_6_: 6-month live weight; LW_12_: 12-month live weight.

## 4 Conclusions

In the present study, the effects of some non-genetic factors on BW, LW_6_, and LW_12_ were statistically significant. Therefore, it is essential to consider these factors (e.g., calving year, calving season, and dam age) in herd management practices. The effect of BW on LL and the effect of LW_12_ on the FCA were statistically significant. The LL of buffaloes with higher BW was longer, and the FCA of buffaloes with higher LW_12_ was shorter. It can be said that buffaloes with higher LW in terms of birth and growth traits had longer LL and earlier FCA. Although it was not statistically significant, LMY increased, and CI tended to decrease with increased LW. As seen in the studies conducted, the number of studies on the effects of BW and growth traits on milk and reproduction traits in buffaloes was limited, and most of the studies were conducted on cattle. Therefore, more studies with large numbers of animals are required to reveal the relationships between related traits in buffaloes.

## Data Availability

The research data from the study are available on request from the corresponding author.

## References

[bib1.bib1] Akbulut Ö, Tüzemen M, Yanar M, Aydın R (1998). Relationships between first lactation milk yield traits and immature body weights and measurements in Brown Swiss cattle. Atatürk University Journal of Agricultural Faculty.

[bib1.bib2] Akkulak Ö, Kul E (2023). Effects of dam milk yield and milk composition on birth weight and growth performance of Anatolian buffalo calves. Buffalo Bull.

[bib1.bib3] Akyol A (2023). Some growth and milk yield characteristics of Anatolian buffaloes and the effect of first calving age and calving interval on these characteristics [Master Thesis].

[bib1.bib4] Alkoyak K, Öz S (2022). The effect of nongenetic factors on calf birth weight and growth performance in Anatolian buffaloes. Turk J Vet Anim Sci.

[bib1.bib5] Atasever S, Erdem H (2008). Water buffalo raising and its future in Turkey. The Journal of Agricultural Faculty of Ondokuz Mayıs University.

[bib1.bib6] Bayou E, Haile A, Gizaw S, Mekasha Y (2015). Evaluation of non-genetic factors affecting calf growth, reproductive performance and milk yield of traditionally managed Sheko cattle in southwest Ethiopia. SpringerPlus.

[bib1.bib7] Boopathi V, Prasad S, Kumaresan A, Manimaran A, Prakash MA (2021). Environmental factors affecting reproductive performance of Murrah buffaloes. Biol Rhythm Res.

[bib1.bib8] Brickell JS, Bourne N, McGowan MM, Wathes DC (2009). Effect of growth and development during the rearing period on the subsequent fertility of nulliparous Holstein-Friesian heifers. Theriogenology.

[bib1.bib9] Carson AF, Dawson LER, McCoy MA, Kilpatrick DJ, Gordon FJ (2002). Effects of rearing regime on body size, reproductive performance and milk production during the first lactation in high genetic merit dairy herd replacements. Anim Sci.

[bib1.bib10] Cooke JS, Cheng Z, Bourne NE, Wathes DC (2013). Association between growth rates, age at first calving and subsequent fertility, milk production and survival in Holstein-Friesian heifers. Open Journal of Animal Sciences.

[bib1.bib11] Eldawy MH, Lashen MES, Badr HM, Farouk MH (2021). Milk production potential and reproductive performance of Egyptian buffalo cows. Trop Anim Health Pro.

[bib1.bib12] Erdem H, Atasever H, Kul E, Önder H, Demirci H (2015). Growth characteristics of anatolian buffalo calves reared in farm conditions: A case study in Samsun province of Turkey.

[bib1.bib13] Ettema JF, Santos JEP (2004). Impact of age at calving on lactation, reproduction, health, and income in first-parity Holsteins on commercial farms. J Dairy Sci.

[bib1.bib14] Göncü S (2020). Cattle breeding: Basic practices in cattle breeding herd management.

[bib1.bib15] Heinrichs AJ, Heinrichs BS (2011). A prospective study of calf factors affecting first-lactation and lifetime milk production and age of cows when removed from the herd. J Dairy Sci.

[bib1.bib16] Hoffman PC (1997). Optimum body size of Holstein replacement heifers. J Anim Sci.

[bib1.bib17] Hossein-Zadeh NG, Madad M, Shadparvar AA, Kianzad D (2012). An observational analysis of secondary sex ratio, stillbirth and birth weight in Iranian buffaloes (Bubalus Bubalis). J Agric Sci Technol.

[bib1.bib18] Koçak S, Tekerli M, Çelikeloğlu K, Erdoğan M, Hacan Ö (2019). An investigation on yield and composition of milk, calving interval and repeatabilities in riverine buffaloes of Anatolia. J Anim Plant Sci.

[bib1.bib19] Kul E, Şahin A, Çayıroglu H, Filik G, Uğurlutepe E, Öz S (2016). Effects of Calving Age and Season on Some Milk Yield Traits in Anatolian Buffaloes.

[bib1.bib20] Kul E, Filik G, Şahin A, Çayıroğlu H, Uğurlutepe E, Erdem H (2018). Effects of some environmental factors on birth weight of Anatolian buffalo calves. Turkish Journal of Agriculture-Food Science and Technology.

[bib1.bib21] López E, Véliz FG, Carrillo E, Santiago ÁD, García JE, Mellado M (2018). Effect of birth weight, weaning weight and preweaning weight gain on fertility of Holstein heifers under hot Mexican conditions. Slov Vet Res.

[bib1.bib22] Marai I, Daader A, Soliman A, El-Menshawy S (2009). Non-genetic factors affecting growth and reproduction traits of buffaloes under dry management housing (in sub-tropical environment) in Egypt. Livestock Research for Rural Development.

[bib1.bib23] Mourits MCM, Huirne RBM, Dijkhuizen AA, Kristensen AR, Galligan DT (1999). Economic optimization of dairy heifer management decisions. Agr Syst.

[bib1.bib24] Mourits MCM, Galligan DT, Dijkhuizen AA, Huirne RBM (2000). Optimization of dairy heifer management decisions based on production conditions of Pennsylvania. J Dairy Sci.

[bib1.bib25] Muasya TK, Peters KJ, Kahi AK (2013). Breeding structure and genetic variability of the Holstein Friesian dairy cattle population in Kenya. Animal Genetic Resources.

[bib1.bib26] Naqvi A, Shami SA (1999). Factors affecting birth weight in Nili-Ravi buffalo calves. Pak Vet J.

[bib1.bib27] Pandya GM, Joshi CG, Rank DN, Kharadi VB, Bramkshtri BP, Vataliya PH, Solanki JV (2015). Genetic analysis of body weight traits of Surti buffalo. Buffalo Bull.

[bib1.bib28] Pirlo G, Miglior F, Speroni M (2000). Effect of age at first calving on production traits and on difference between milk yield returns and rearing costs in Italian Holsteins. J Dairy Sci.

[bib1.bib29] Sadek R, Ashour G, Ibrahim MA, Samoul AM (2014). Effect of daily weight gain on age at first calving and subsequent milk yield of Holstein heifers in Egypt. Egyptian Journal of Animal Production.

[bib1.bib30] Segura-Correa JC, Magaña-Monforte JG, Aké-López JR, Segura-Correa VM, Hinojosa-Cuellar JA, Osorio-Arce MM (2017). Breed and environmental effects on birth weight, weaning weight and calving interval of Zebu cattle in Southeastern Mexico. Tropical and Subtropical Agroecosystems.

[bib1.bib31] Serjsen K, Garnsworthy PC (2005). Calf and Heifer Rearing: Principles of Rearing the Modern Dairy Heifer from Calf to Calving.

[bib1.bib32] Sorathiya LM, Fulsoundar AB, Kharadi VB (2009). Environmental and genetic effects on body weight in Surti buffalo calves. Indian J Anim Sci.

[bib1.bib33] Soysal Mİ, Genç S, Aksel M, Ünal EÖ, Gürcan EK (2018). Effect of environmental factors on lactation milk yield, lactation length and calving interval of Anatolian buffalo in Istanbul. Journal of Animal Science and Products (JASP).

[bib1.bib34] Tekerli M (2015–2018). Buffalo Star, Data Recording, Accounting and Project Tracking Program. Afyon Kocatepe University.

[bib1.bib35] Thiruvenkadan AK, Panneerselvam S, Rajendran R (2009). Non-genetic and genetic factors influencing growth performance in Murrah buffalos. S Afr J Anim Sci.

[bib1.bib36] Tozer PR, Heinrichs AJ (2001). What affects the costs of raising replacement dairy heifers: A multiple-component analysis. J Dairy Sci.

[bib1.bib37] Turkish Statistical Institute (2024). TUIK.

[bib1.bib38] Uğurlu M, Kaya I, Saray M (2016). Effects of some environmental factors on calf birth weight and milk yield of Anatolian Water Buffalo (Bubalus bubalis). Bulg J Agric Sci.

[bib1.bib39] Verma R, Vijayalakshmi K, Virmani M, Kumar S, Verma A (2021). Seasonal influence of age at first calving on genetic variation and subsequent reproductive performances in Murrah buffaloes. Biol Rhythm Res.

[bib1.bib40] Wathes DC, Brickell JS, Bourne NE, Swali A, Cheng Z (2008). Factors influencing heifer survival and fertility on commercial dairy farms. Animal.

[bib1.bib41] Yadav BS, Yadav MC, Singh A, Khan FH (2001). Murrah buffaloes-1. Birth weight. Buffalo Bull.

[bib1.bib42] Yıldız N, Aygen S, Özçelik M (2008). Milk production, reproductive and body measurements in Eastern Anatolian Red cows reared at Elazığ situations. ii. Milk production traits, body measurements, body weight, calf birth weight and survivality of Eastern Anatolian Red cows reared at Elazığ situations. Fırat University Veterinary Journal of Health Sciences.

[bib1.bib43] Yüceer B, Özbeyaz C (2010). Effect of immunity on growth, disease incidence and livability in calves after colostrum consuming. Ankara Univ Vet Fak.

